# SpecRep: Adversary Emulation Based on Attack Objective Specification in Heterogeneous Infrastructures

**DOI:** 10.3390/s24175601

**Published:** 2024-08-29

**Authors:** Radu Marian Portase, Adrian Colesa, Gheorghe Sebestyen

**Affiliations:** 1 Computer Science Department, Technical University of Cluj Napoca, 400114 Cluj Napoca, Romania; 2 Bitdefender, 060071 Bucharest, Romania

**Keywords:** adversary emulation, attacks against complex infrastructures, cyber security for the smart city, formal languages, large language models used for knowledge extraction

## Abstract

Cybercriminals have become an imperative threat because they target the most valuable resource on earth, data. Organizations prepare against cyber attacks by creating Cyber Security Incident Response Teams (CSIRTs) that use various technologies to monitor and detect threats and to help perform forensics on machines and networks. Testing the limits of defense technologies and the skill of a CSIRT can be performed through adversary emulation performed by so-called “red teams”. The red team’s work is primarily manual and requires high skill. We propose SpecRep, a system to ease the testing of the detection capabilities of defenses in complex, heterogeneous infrastructures. SpecRep uses previously known attack specifications to construct attack scenarios based on attacker objectives instead of the traditional attack graphs or a list of actions. We create a metalanguage to describe objectives to be achieved in an attack together with a compiler that can build multiple attack scenarios that achieve the objectives. We use text processing tools aided by large language models to extract information from freely available white papers and convert them to plausible attack specifications that can then be emulated by SpecRep. We show how our system can emulate attacks against a smart home, a large enterprise, and an industrial control system.

## 1. Introduction

Our daily lives are critically linked to computers and the internet. We shop online, handle our taxes and banking online, and even interact with our families online. The rapid growth of the Internet of Things (IoT) and its applications draws the attention of advanced cyber adversaries towards the smart city and the smart home [1]. The security risks of the current rapid adoption of digital technology can be devastating. A cyber attack can cripple the power grid, disrupt all traffic, whether by land, sea, or air, and even trigger critical failures in nuclear power plants. Democracy itself can become a target of hackers. It is well-known that the most powerful state armies have cyber divisions that develop and defend against cyber weapons. The current global context increasingly compels researchers and defenders to understand how advanced malicious actors operate, to study their tools, techniques, and procedures (TTPs), and to defend infrastructures against attacks.

Large-scale public and private organizations employ Cyber Security Incident Response Teams (CSIRTs) to receive, review, and respond to information security incidents. The scope of CSIRT activities can vary widely: they might monitor a corporate organization, a military organization, an educational institution, the infrastructure of a city, or even an entire country.

Organizations improve the efficiency of their CSIRTs using offensive testing. Penetration testing reveals an organization’s vulnerabilities and how they might be compromised. The same ethical hacking processes can help improve security for IoT and smart cities [2].

Adversary emulation, or red teaming, involves simulating actual end-to-end attacks by applying TTPs from advanced real-world scenarios. Red teams consist of highly skilled individuals who create and execute attack scenarios manually [3,4]. These individuals usually perform one of two duties. First, they help organizations continuously test and validate the effectiveness of their cybersecurity defenses. They assess both prevention and detection controls, allowing organizations to identify vulnerabilities and optimize their security posture. This activity is conducted directly on the enterprise network that is tested. Second, they test the detection and prevention capabilities of endpoint protection platforms (EPPs) and endpoint detection and response (EDR) solutions and try to find ways to bypass them. This second activity is usually performed inside an adversary emulation environment of virtual and real machines protected by the tested solution.

Traditional attack emulation tools use attack graphs to model vulnerabilities in networked systems through a series of discrete exploits that lead to a compromised security state [5]. Hybrid attack graphs are more suitable for modeling the discrete (cyber) and continuous (physical) components of cross-domain cyber-physical attacks [6]. From a blue team perspective, attack graphs are easy to follow and understand. Applying game theory to the analysis of attack graphs enhances network security [7]. From a red team perspective, attack graphs can offer insights on how to plan an attack on a network [8].

Existing work on adversary emulation focuses on the actions performed by the red team on a machine. Typically, execution follows a fully specified scenario as a set of exact steps that are performed either by hand or by some execution engine. The exact details of each step are hardcoded in the scenario. This severely limits the coverage of test cases when evaluating the detection and visibility of a security solution and does not replicate how human adversary would act. A human adversary, when blocked by a security solution, will try alternate ways to achieve the same desired result. In this paper, we propose a shift in perspective, focusing on the objectives that need to be achieved. We transition from traditional attack graphs to a view based on objective graphs and attack stages.

We propose an architecture for an adversary emulation system named SpecRep. Our system employs a metalanguage derived from the MITRE ATT&CK matrix [9] to describe an attack as a series of attacker objectives to be achieved, rather than following the traditional graph of attack steps. Internally, SpecRep can achieve each objective in multiple ways using open-source and internally developed tools. Our design is easily extendable and allows us to integrate new tools in existing attack scenarios. The creation and integration of new tools is an ongoing process.

A complete test run using SpecRep emulates all possible variations to achieve all specified objectives. This approach enables the comprehensive testing of security solutions, as it accounts for the diverse methods that adversaries might interchangeably use to achieve their goals. By focusing on attacker objectives rather than specific actions, SpecRep provides a more flexible and scalable framework for evaluating the EPP and endpoint detection and response EDR solutions against advanced persistent threats (APTs).

The main contributions of our work are as follows:

- We propose an innovative approach to describe cyber attacks as a series of attacker objectives rather than relying on traditional attack graphs or lists of attacker actions;
- We create a specialized metalanguage to describe attacker objectives clearly;
- We compile files written in this objective specification metalanguage into multiple attack specifications that contain many variations of actions that lead to the achievement of the specified objectives;
- We create a system that utilizes large language models to convert freely available white papers to attack objectives.

The structure of this paper is organized as follows. In Section 2, we review the existing literature on adversary emulation. In Section 3, we describe our system’s architecture, including the components of the SpecRep metalanguage files and our attack emulation framework. In Section 4, we showcase how our system can be used to emulate attacks against a home user, an enterprise organization, and an industrial controls system. We discuss the implications of our work in Section 5 and propose some future work objectives in Section 6. Finally, Section 7 provides the conclusion of this paper.

## 2. Related Work

Adversary emulation was established as a research subject at the end of the 1990s. The authors started by creating taxonomies for attackers, attack types, defense mechanisms, and the impact of attacks. Cohen’s work on the subject of the classification of attacks on information systems [10] and simulation of attacks on information systems [11] is an excellent example in this direction. Later, researchers focused on determining attack paths in enterprise networks and simulating them. Park et al. continued Cohen’s early work by developing SECUSIM [12], a simulator able to define attack mechanisms, confirm defense strategies, and analyze the attack’s effects. Both Cohen and Park modeled and implemented attacker behavior as very high-level events and did not generate or test sensor output.

A notable early example of an attack emulation system is Kotenko et al.’s “Attack Simulator” [13]. The authors used probabilistic step machines defined in high-level attack steps like Enumeration, Privilege Elevation, and Backdoor Creation to generate attacks. These steps may be considered precursor names to the now-established MITRE ATT&CK taxonomy [9]. We also model attacks as high-level attack steps but focus on the attacker’s goal instead of the actions performed.

Current work on adversary emulation acknowledges the advantages of using MITRE ATT&CK for adversary emulation. Ajmal et al. [14] introduced a threat-based adversary emulation approach that enhances traditional penetration testing and red teaming by providing realistic simulations of real-world attacks. Utilizing the Mitre ATT&CK framework, the approach continuously adapts to an organization’s evolving security posture, mimicking advanced persistent threats (APTs) and sophisticated attackers. We also use the freely available knowledge from Mitre ATT&CK to generate realistic attack scenarios.

The authors of [15] investigated the use of deep reinforcement learning (DRL) to enhance adversarial cyber-attack simulations and improve cybersecurity. Traditional ML-based security applications, often limited by historical data and generalizability, are contrasted with AI-assisted red teaming, which uses DRL to teach autonomous agents optimal attack strategies in complex networks. SpecRep can help with the training of adversarial agents because our approach allows us to generate all known possibilities to achieve an attacker’s objective.

Today, several tools for automated adversary emulation already exist. CALDERA [16], created by Applebaum et al. [17], is the attack emulation framework popularized and owned by the MITRE corporation. The framework automatically attacks target systems using tactics and techniques described in the ATT&CK framework [9]. A sister project named CASCADE [18] will possibly automate investigative work after an attack is emulated. We also use the ATT&CK matrix, but, unlike CALDERA, we focus on automatic tests rather than helping manual red teams. Our automatic approach can vastly increase the test surface.

Atomic Red Team [19] offers simple atomic tests that exercise the same techniques used by adversaries. The tests are mapped to the MITRE ATT&CK matrix. Our system uses actions that can be compared to atomic tests, but the actions are executed as a part of a full attack emulation, not as individual parts.

The APTSimulator [20] is an adversary emulation tool that is designed to be very simple. It makes a system appear to be compromised by using a Windows Batch script that calls a set of tools and output files. Unlike the APTSimulator, our system generates a full attack kill chain.

Various companies offer tools that are categorized as Breach and Attack Simulation (BAS) Tools [21]. These tools run automated tests against infrastructures to test the security posture vulnerabilities of a company. Our SpecRep system can be categorized as a BAS Tool that can automatically generate new attack flows starting with white papers or threat intelligence reports instead of simply relying on a predefined portfolio of simulated attacks.

## 3. Materials and Methods

In this section, we detail the design and development of our SpecRep system. The initial iteration of this system aimed to replicate cyber attacks within a virtual lab environment. Its primary objective was to evaluate endpoint protection platforms (EPPs) and endpoint detection and response (EDR) solutions against advanced persistent threats (APTs). Initially, the system required analysts to craft Python scripts to execute each step of an attack scenario and manually verify the output of the security solution under test. Each script was tailored to a specific type of attack, with actions performed sequentially and precisely defined. These Python scripts essentially functioned as state machines, following an attack graph to replicate the specified attack steps.

A significant challenge with adhering strictly to an attack scenario graph is that a single attack objective can often be achieved through numerous methods. For instance, attackers seeking to register a program to start upon system reboot in Windows have over 100 different methods using Registry keys alone [22]. Creating and implementing scripts to reproduce all these variations would be impractical. To address this issue, we shifted our approach from focusing on “how an attacker acts” to “what objectives an attacker aims to achieve.” This perspective shift led us to concentrate on attacker objectives rather than the specific methods used to achieve them.

By adopting this objective-based approach, we enhanced the flexibility and efficiency of the SpecRep system, enabling it to more effectively emulate a wide range of attack scenarios without the need for an exhaustive number of scripts. This strategic pivot allowed for a more comprehensive and scalable testing framework for assessing the robustness of EPP and EDR solutions against evolving cyber threats.

### 3.1. Emulation System Architecture

**Figure 1:**
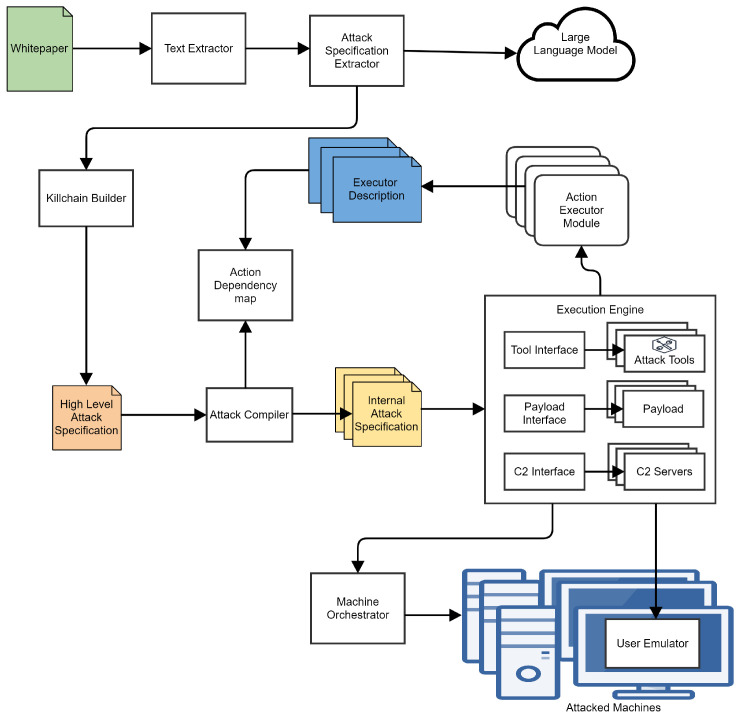
SpecRep system architecture.

We built the current generation of SpecRep around the concept of describing attacker objectives and implementing multiple ways in which they could be achieved. The architecture and flow of SpecRep is presented in Figure 1.

High-Level Attack Specifications (HLASs) are the embodiment of our objective-focused view of attack emulation. We write specifications in specification files to describe attacks (with a custom-made metalanguage) as objectives to be achieved. Our system can be split into three distinct parts concerning high-level attack specifications.

The first part of the system is responsible for processing information about cyber attacks in the form of freely available white papers and translating them to HLASs. We start the conversion using text extractors that can process various white paper formats (e.g., HTML, PDF, etc.) and extract relevant text from them. The Text Extractor has some rough logic to skip certain parts of white papers based on simple heuristics like names of chapters or missing keywords in text. We continue the HLAS extraction process by using OpenAI API [23] to extract information from the white paper that is as close to MITRE tactics and techniques as possible. Our Attack Specification Extractor normalizes the output received from Open AI and extracts all possible attacker objectives together with details about how they were achieved. Because a white paper may present more than one instance of an attack, we use a Killchain Builder component that extracts one or more plausible attacks and generates High-Level Attack Specifications from them.

The second part of the system is responsible for compiling HLASs into an Internal Attack Specification (IAS) format that can be executed against real or virtual infrastructure. The Attack Compiler reads a high-level specification and creates all combinations of known actions that can be used to achieve the specified objectives. Known actions (i.e., actions that have an implementation) are described in Executor Description files.

The final part of the system runs the attack against Test Machines that may be monitored by a tested security solution. The *Execution Engine* reads an internal specification and begins executing it. The engine executes attacks using a wide range of open attack tools (e.g., Metasploit [24], Powershell Empire [25]) or custom tools created by us. During execution, multiple payloads built using various programming languages (e.g., C++, JavaScript, Visual Basic Script) may be deployed and used. Communication between the execution engine and the payloads may be performed using different sets of command and control (C2) clients and servers. Each action available to the execution engine is implemented inside an Action Executor Module. Actions that require user interaction such as opening an e-mail may be executed by a User Emulator agent deployed on some of the tested machines.

### 3.2. Formalized Attack Specification

For our high-level event specification, we use metalanguage to describe an attack. A simplified form for the grammar of the metalanguage is presented in Listing 1.

**Listing 1.** The SpecRep metalanguage grammar.

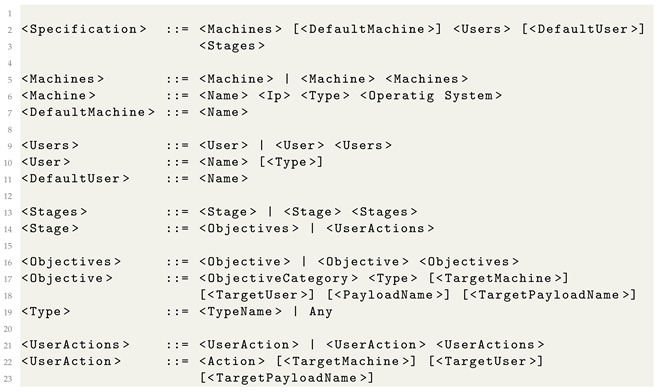



The *Machines* section of the metalanguage file describes all the machines involved in an attack. Multiple important properties can be set for each machine. An example would be the machine’s IP address, its type (be it an IoT device like a router, a computer, or even an industrial control system), or the operating system. An optional *DefaultMachine* field establishes the name of the default machine on which to run actions unless otherwise specified.

The *Users* section describes each user who will perform actions during a scenario’s execution. Users have different roles inside the adversary emulation environment and belong to different groups inside an active directory. An optional *DefaultUser* field sets the name of the default user that performs actions unless otherwise specified.

The *Stages* section models the attack timeline imposed by the different dependencies between objectives in an attack. For example, obtaining access to a machine must be completed before an attacker registers persistence; we model initial access as stage 1 and setting persistence as stage 2.

In SpecRep, we define a series of objectives that an attacker may aim to achieve at each stage of an attack. If a stage includes multiple objectives, the sequence in which they are executed is not crucial; the compiler will generate specifications for all possible permutations of the objectives within that stage. For example, if Stage I contains objectives O1 and O2 and Stage II contains objectives O3 and O4, the compiler will generate scenarios for the following permutations:

- {O1, O2, O3, O4};
- {O1, O2, O4, O3};
- {O2, O1, O3, O4};
- {O2, O1, O4, O3}.

The compiler ensures that objectives from different stages are not mixed; thus, a scenario such as {O1, O3, O2, O4} will never be produced.

Each objective has a category and a type:

- ObjectiveCategory: This represents the high-level goal that an attacker wants to achieve, such as gaining access to a machine or obtaining persistence;
- Type: This is an internal name for the specific implementation within SpecRep that achieves the given objective.

Some objectives conclude with deploying a specific payload to a target computer. This payload acts as a remote access trojan, retrieving commands from our command and control server. We use *PayloadName* to assign a name to a newly delivered payload. We can then instruct the system to use this payload to achieve other objectives by specifying *TargetPayloadName* for the objective.

Additionally, the objectives can specify the machine and user under which the code should be executed through the optional fields *TargetMachine* and *TargetUser*. If these fields are not provided, the system defaults to the *DefaultMachine* and *DefaultUser* specified in the attack specification.

The *UserActions* field of a stage contains all actions that a user executes during the said stage. Example actions include rebooting the computer, opening and clicking a link in an email, or running a specific program received as an attachment. Similar to the objectives, we can specify the *TargetMachine*, *TargetUser*, or *TargetPayloadName*.

An example of attack objective specification is presented in Listing 2. The attack uses a single machine and a single user. The attacker first wants to achieve initial access, then register persistence and wait for the user to reboot the computer. Processing this specification through the Attack Compiler will generate all combinations of Initial Access + Persistence known by the SpecRep system because we specified *“Type”: “Any”*. The compiler adds multiple sub-stages and actions during compilation when creating the internal attack specifications. For example, initial access using a spearhead phishing e-mail requires the attacker to send the e-mail and the user to open the e-mail, click on the phishing link, download a payload, and execute it.

**Listing 2.** Example of a high-level attack description.

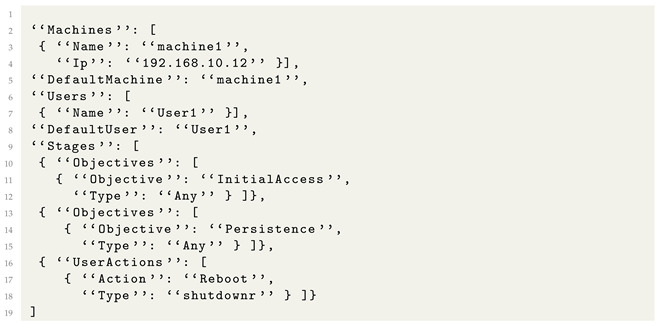



### 3.3. Compiling Metalanguage to Attack Specification

As previously stated, the Attack Compiler transforms high-level metalanguage attack objective specifications into Internal Attack Specifications. Because Internal Attack Specifications are interpreted by the *Execution Engine*, the compiler needs to know all actions that the execution engine can perform. Each action that can be executed has an *executor description file* that describes a series of dependencies on other actions, the artifacts produced, and a description of expected events. This file is a JSON with the following important fields:

- Objective: The objective that is achieved when this action is performed;
- Type: Type identifier for this objective;
- ImplementationId: An ID for the action executor module. SpecRep supports multiple implementations for the same Objective, Type pair;
- DependsOn: Array of dictionaries with entities that are dependencies for the current action. Each system entity has a *Type* and a *Name*;
- Provides: Array of entities that are produced in the system when this action is executed.

Our Attack Compiler relies on two important algorithms for its execution. We present their simplified forms below.

The compiler builds an action dependency map using Algorithm 1. The action dependency map is a directed graph created based on executor descriptions. Vertices in the graph are added based on relations between DependsOn and Provides.

The compilation itself (Algorithm 2) is based on pathfinding in the action dependency map. The compiler reads a high-level attack specification, selects a possible end node from it, and makes a list of constraints that can be deduced based on the stages. The constraints are the objective nodes that need to be reached for each attack stage and the order between stages. The compilation then uses a custom implementation of depth-first search to generate all paths in the graph that leads from the start node to the end node and respects the constraints. For each path, the compiler will output an Internal Attack Specification.
**Algorithm 1** Build Action Dependency Map  1:ActionDependencyMap←newDirectedGraph  2:startNode←newDirectedGraphNode  3:ActionDependencyMap.insert(startNode)  4:ExecutorDescriptions←newList  5:**for** description in executorDescriptionFiles **do**  6:    executor←LoadJson(description)  7:    ExecutorDescriptions.append(executor)  8:**end for**  9:**for** executor in ExecutorDescriptions **do**10:    action←newDirectedGraphNode(executor)11:    ActionDependencyMap.insert(action)12:**end for**13:**for** executor in ExecutorDescriptions **do**14:    executorNode←ActionDependencyMap.find(executor)15:    **for** artefact in $executor[“Provides^{”}]$ **do**16:        **for** description in ExecutorDescriptions **do**17:           **if** description≠executor **and** artefact in $description[“DependsOn^{”}]$ **then**18:               dependsNode←ActionDependencyMap.find(description)19:               ActionDependencyMap.addEdge(executorNode,dependsNode)20:           **end if**21:        **end for**22:    **end for**23:**end for**24:**return** ActionDependencyMap

**Algorithm 2** Compile High-Level Attack Description
  1:

ActionDependencyMap←BuildActionDependencyMap()

  2:

StartNode←ActionDependencyMap.getStartNode()

  3:

AttackDescription←LoadJson(pathtohigh-levelattackdescription)

  4:

StageSuccession←newList

  5:**for** stage in $AttackDescription[“Stages^{”}]$ **do**  6:    stageNodes←newList  7:    **for** objective in $stage[“Objectives^{”}]$ **do**  8:        node←ActionDependencyMap.find(objective)  9:        stageNodes.append(node)10:    **end for**11:    StageSuccession.append(stageNodes)12:
**end for**
13:

EndNode←ExtractEndNode(StageSuccession)

14:

Constraints←ExtractConstraints(StageSuccession)

15:

ValidPaths←DepthFirstSearchWithConstraints(StartNode,EndNode,Constraints)

16:**for** path in ValidPaths **do**17:    Initialize InternalAttackSpecification18:    **for** node in path **do**19:        InternalAttackSpecification.addDescriptions(node)20:    **end for**21:    Write InternalAttackSpecification22:
**end for**



Consider the following example of compilation. An attacker wants to perform user discovery on any machine available. The infrastructure has two machines. A reduced version of SpecRep implements only the following capabilities:

- Port scan using nmap utility to find machines vulnerable to Eternal Blue (Listing 3);
- Metasploit exploitation of Eternal Blue that executes Meterpreter payload (Listing 4);
- Metasploit exploitation of Eternal Blue that executes cmd.exe (Listing 5);
- Discovery current user using whoami form cmd.exe (Listing 6);
- Discovery current user Meterpreter payload (Listing 7);
- Deploy a Koadic payload (Listing 8);
- Use Koadic to perform the lateral movement (Listing 9).

**Listing 3.** Port scan executor description.

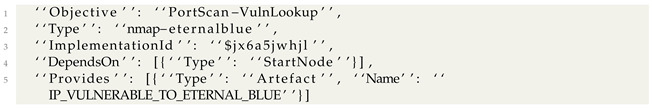



**Listing 4.** Initial access via Meterpreter executor description.

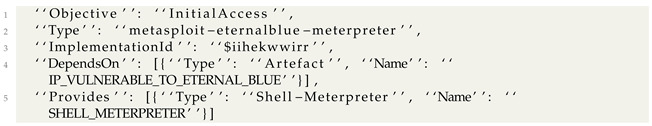



**Listing 5.** Initial access via CMD executor description.

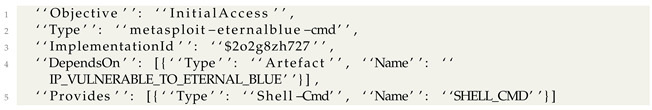



**Listing 6.** Discovery: User via CMD executor description.

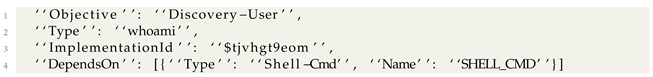



**Listing 7.** Discovery: User via Meterpreter executor description.

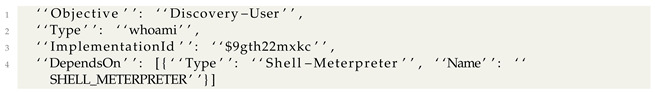



**Listing 8.** Deploy Koadic payload executor description.

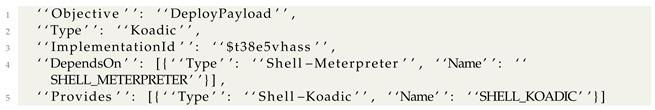



**Listing 9.** Lateral movement via Koadic executor description.

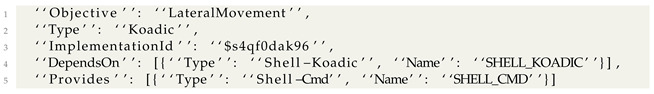



**Figure 2:**
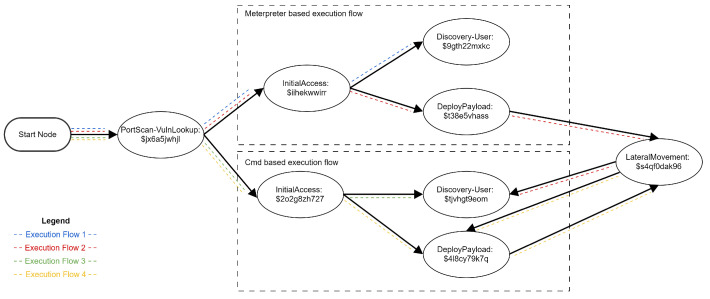
Simplified action dependency map.

Based on these files, the attack compiler can build the actions’ dependency map, as shown in Figure 2. Please notice how, after lateral movement, we can deploy Koadic on the target machine or perform user discovery.

We can specify that an attacker wants to perform user discovery in various degrees of detail starting with a description of a full flow (Listing 10) and finishing with just an objective (Listing 11). The attack compiler follows the Action Dependency Graph and generates attack flows based on the content of the High-Level Attack Specification. The detailed description will generate a single possible attack flow: Execution Flow 1 from Figure 2. Specifying the single user discovery objective will generate four attack flows, as seen in the same figure, i.e., Figure 2.

**Listing 10.** Example of detailed attack flow description.

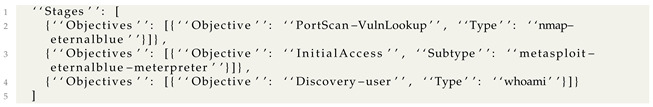



**Listing 11.** Example of just one objective to be achieved.

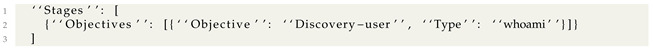



### 3.4. Execution of Internal Attack Specifications

After compilation, the Attack Compiler generates a series of internal attack specification files that contain all the necessary details to emulate an attack. These files guide the execution engine, specifying the precise actions to take, the sequence in which to perform them, and the machines involved. Once these details are set, the Machine Orchestrator starts the relevant machines, and the attack can proceed. All machines managed by the orchestrator have the User Emulator pre-installed, ready to execute commands if user interaction is part of the attack scenario.

Execution is the phase where the robustness of Endpoint Protection Platforms (EPPs) and Endpoint Detection and Response (EDR) solutions against evolving cyber threats are tested. The target machines can be configured to run the security solution being evaluated. The execution engine performs all the attack scenarios outlined in the internal specifications, noting which objectives are achieved in each variation. Alternatively, the security solutions can be set to a non-blocking mode, allowing all attack objectives to be met. Any gaps in the visibility of the tested solution can be identified by parsing the security solution’s logs and comparing them with the actions executed by the engine.

## 4. Results

### 4.1. Attack Specification Extraction

We tested our High-Level Attack Specification extractor using known actors described in the MITRE pages about threat actors [26] and some of their bibliographies. We selected test examples to cover the three main classes of attacks that can be emulated by our system as follows.

First, we selected 11 threat actors that are proactive in targeting large organizations. Next, we gathered information about IoT botnets that target household appliances and routers. Finally, we included attacks that target industrial control systems.

We successfully generated 56 attack specifications from these sources. A breakdown of all results is shown in Table 1.

### 4.2. Attack Emulation Examples

In this section, we discuss simplified examples of attacks generated by our system to showcase how SpecRep can be used to test and improve the security posture of a smart home, a large organization, and an industrial control system. We delve into implementation details and highlight some advantages of our approach over others.

We perform our tests on virtual infrastructure, replacing hardware components that were targeted in attacks with virtual machines that emulate similar behavior. This allows us to simulate real-world attack scenarios in a controlled and repeatable environment.

### 4.3. Attack against a Smart Home Inspired by Mirai IoT Botnet

Mirai is one of the most well-known families of Internet of Things (IoT) botnet malware. The source code for the malware was leaked in 2016 and is easily available to malicious actors. Recent articles, such as [27], show how threat actors use a multitude of IoT vulnerabilities to spread slightly modified variants of the original botnet code.

**Figure 3:**
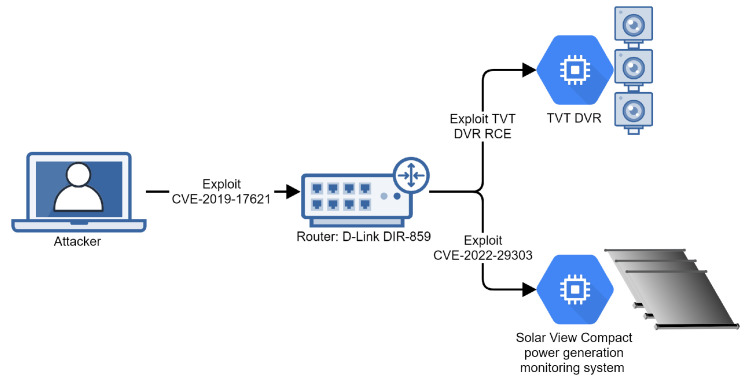
Infrastructure for Mirai emulation.

Our emulation for Mirai targets a smart home (Figure 3) that uses a D-Link DIR-859 router to connect to the internet. The home is monitored by a series of webcams connected to a TVT DVR and is powered by a series of solar panels monitored via the Solar View Compact power-generation monitoring system. According to [27], attackers deploying Mirai are known to be able to exploit all of these devices.

Our emulation runs on four virtual machines. The first machine emulates the attacker, a second small Linux machine emulates the D-Link DIR-859 router (vulnerable to CVE-2018-17621: Unauthenticated Remote Command Execution [28]), and two other small Linux machines emulate the TVT DVR controller (vulnerable to Remote Code Execution [29]) and the Solar View Compact power-generation monitoring system (vulnerable to CVE-2022-29303: Command Injection [30]).

The Linux machines were created to have the same vulnerabilities seen exploited in the wild. They run specific Python programs that can be exploited by the proof of concept scripts for the previously mentioned vulnerabilities.

The emulation plan follows the actions of a typical Mirai threat actor in the market:

- Stage 1. The attacker performs a port scan to find a host vulnerable to CVE-2019-1721;
- Stage 2. The attacker exploits CVE-2019-1721 and obtains a shell of the router;
- Stage 3. The attacker performs another port scan, to find hosts vulnerable to CVE-2022-29303;
- Stage 4. The attacker compromises the solar panel server using CVE-2019-1721;
- Stage 5. The attacker deploys a shell on CVE-2022-29303;
- Stage 6. The attacker performs a port scan to find hosts vulnerable to the TVT DVR exploit;
- Stage 7. The attacker exploits the DVR system;
- Stage 8. The attacker deploys a shell on the DVR system.

After the emulation is complete, the emulated actor has added the router, dvr system, and solar panel management server to the botnet.

The simplified emulation metalanguage for the attack is presented in Listing 12. The same shell named “SpecRep-shell-linux” is used in the “PayloadName” and “TargetPayloadName” fields to specify exactly which shells are used when performing actions. Notice that each machine will have two shells open during the execution. The first shell is obtained after exploiting a vulnerability for initial access, and the second shell is explicitly deployed to simulate that the machines were added to a botnet. If we do not specify one or some of the “TargetPayloadName” fields, the SpecRep compiler would generate multiple attack flows to utilize the various shells available. The simulated attacker might even try to attack the DVR or the solar panel servers directly.

**Listing 12.** High-level attack description for Mirai.

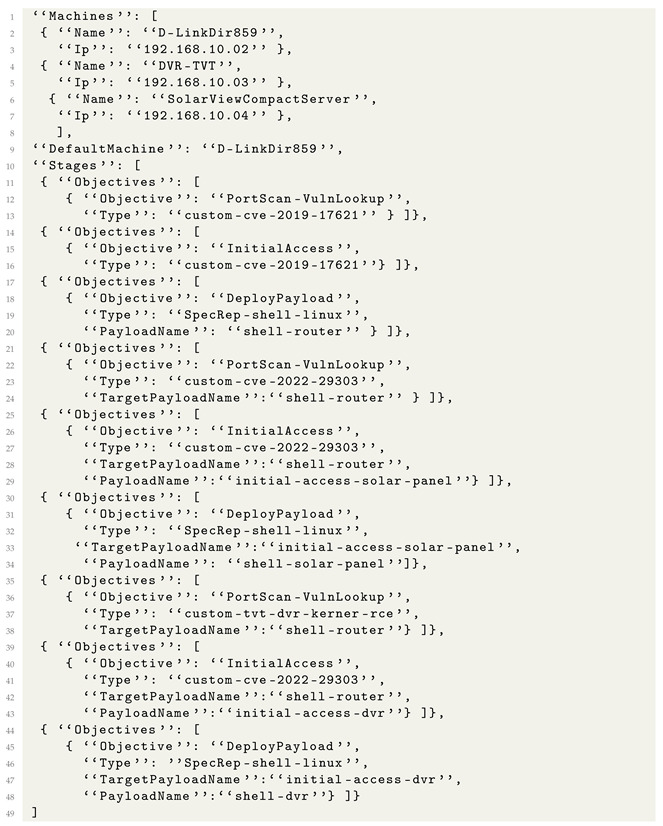



### 4.4. Attack against a Large Enterprise Inspired by APT28 (Fancy Bear)

APT28 [31], also known as STRONTIUM [32], Sednit [33], Sofacy [34], and Fancy Bear, is one of the threat groups attributed to Russia’s General Staff Main Intelligence Directorate (GRU) [35].

We created an adversary emulation description for APT28 based on some of the threat actors’ techniques when interfering with the U.S. presidential election in 2016 [36].

**Figure 4:**
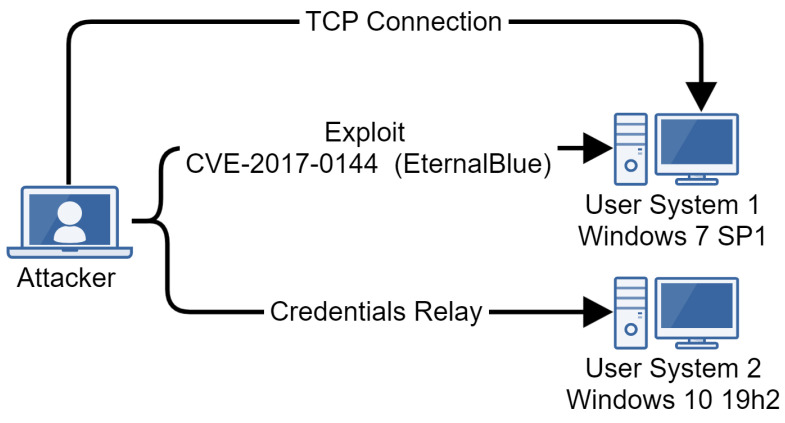
Infrastructure for APT28 emulation.

Our emulation runs on two machines (Figure 4), each with a version of Windows 7 Service Pack 1 installed and added to the active directory. The goal of the emulation is to obtain access to one of the machines via the exploitation of CVE-2017-0144 [37], known as EternalBlue, perform some discovery actions, deploy a post-exploitation framework named Koadic [38], obtain persistence, and laterally move to the second machine using a technique known as credentials relay [39].

The emulation specification for APT28 is created as follows:

- Stage 1. The attacker performs a port scan to find a host vulnerable to CVE-2017-0144;
- Stage 2. The attacker exploits CVE-2017-0144 and obtains a shell of the target;
- Stage 3. The attacker performs a list of discovery actions on the target;
- Stage 4. The attacker deploys a Koadic payload on the target;
- Stage 5. The attacker uses Koadic to register persistence and deploy a Responder payload on the target;
- Stage 6. The attacker moves laterally to the second target machine;
- Stage 7. The attacker runs discovery commands on the second target machine;
- Stage 8. The attacker performs cleanup on the first target machine.

The emulation metalanguage file for APT28 is presented in Listing 13. Because we strongly specified each objective using the *Type* entries, when the emulation metalanguage file is compiled, we obtain some precise attack flows, where we know exactly which tools and techniques are used for each stage of the attack. This assures that the execution engine will perform attacks that remain true to the TPPs used by the modeled attack, even if they contain small alterations.

SpecRep automatically creates multiple variations starting from the metalanguage objective specification of APT28. The first source of variation comes from the order in which objectives in stages 3 and 5 are executed. The second and more significant variation source comes from how the Koadic payload is deployed. By design, Koadic supports many stagers (ways to execute the framework payload on the compromised machine). We believe that changing the stager is one of the first things that an attacker would try in order to bypass a security solution. In the current implementation of SpecRep, a construct such as *{“Objective”: “DeployPayload”, “Type”: “koadic”}* translates to five different actions, each with the same end result of deploying Koadic, but using a “bitsadmin”, “mshta”, “regsvr32”, “rundll32”, or “wmic” to run a Koadic stager. If we implement a new way to deploy Koadic on the machine, the APT28 scenario will be updated simply by recompiling the attack. This architectural choice for SpecRep tests that a security solution detects an attack even with small variations in how the attack objectives are achieved.

The SpecRep metalanguage allows a user to specify commands for a payload by matching the PayloadName and TargetPayloadName. For example the compiler matches stage 4 *{“Objective”: “DeployPayload”, “Type”: “koadic”, “PayloadName”: “koadic-1”}* with stage 5 *{“Objective”: “Persistence”, “Type”: “WMI”, “TargetPayloadName”:“koadic-1”}* and uses the Koadic framework to register persistence using WMI.

**Listing 13.** High-level attack description for APT28.

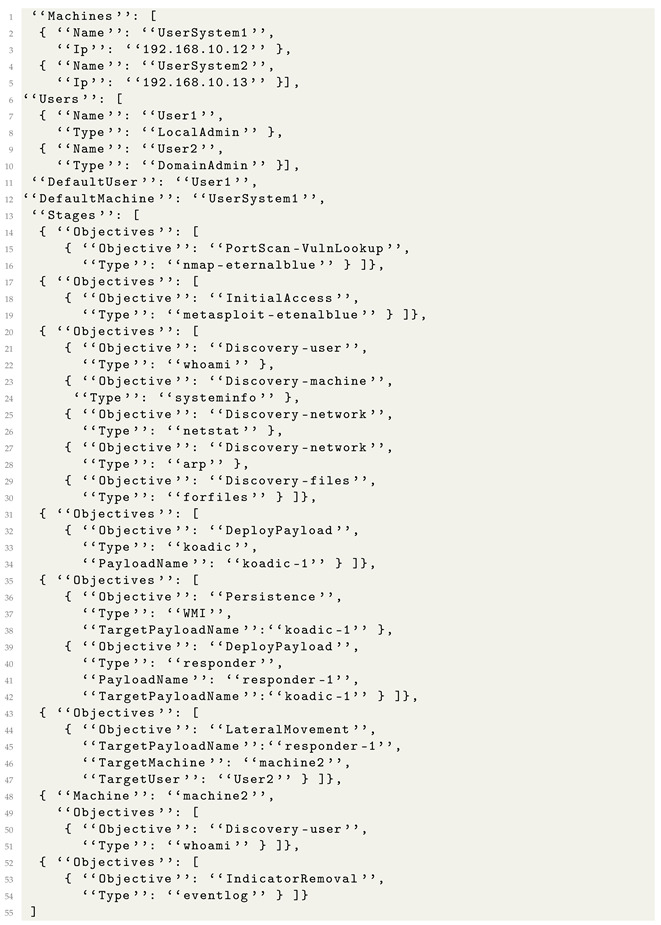



Finally, as previously stated, the SpecRep compiler has a built-in mechanism that is capable of inserting required UserActions automatically. For the stage 6 objective *{“Objective”: “LateralMovement”, “TargetPayloadName”: “responder-1”, “TargetMachine”: “machine2”, “TargetUser”: “User2”}*, in order for the credential relay to function, it requires the target user to perform an SMB login; the compiler knows this and inserts a user action to login as User2 on machine1. The user actions are executed by the previously mentioned *UserEmulator* program.

### 4.5. Attack against an Industrial Control System Inspired by Chernovite Badomen

In 2022, Dragos, a cybersecurity company, reported that it had identified and analyzed a new Industrial Control Systems-specific malware, which they named PIPEDREAM [40]. Their report presents several attack tools designed to disrupt or potentially destroy industrial environments, specifically ones using hardware produced by Omron and Schneider. We created an emulation plan based on their report to specifically target an Omron NX1P2 connected to an operator PC and a servo motor.

**Figure 5:**
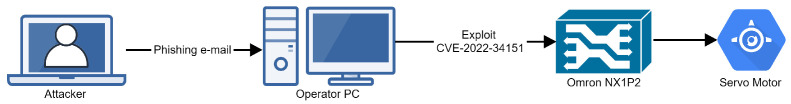
Infrastructure for Chernovite Badomen emulation.

The network diagram for the infrastructure that we created is presented in Figure 5. We used a Windows virtual machine as an Operator PC and a Linux virtual machine that emulates the software present on Omron NX1P2 and that is vulnerable to CVE-2022-34151. We created the Omron machine based on [41].

Our emulation plan for Badomen assumes that an operator was compromised via a phishing e-mail. The stages of the attack are as follows:

- Stage 1. The attacker gains initial access to the operator machine using a spear-phishing attachment executable;
- Stage 2. The attacker scans the network for machines vulnerable to CVE-2022-34151;
- Stage 3. The attacker connects to the Omron machine by exploiting CVE-2022-34151;
- Stage 4. The attacker changes parameters for the servo motor emulated to be connected to the Omron machine.

A simplified emulation metalanguage file is presented in Listing 14. The *UserEmulator* component of SpecRep knows how to simulate the phishing attack by downloading the attachment and running it without explicit specification. We only emulate a small change to the servo motor, as suggested in [41]. More complicated changes would require us to extend our emulator software for the Omron device or to use a real device.

**Listing 14.** High-level attack description for Badomen.

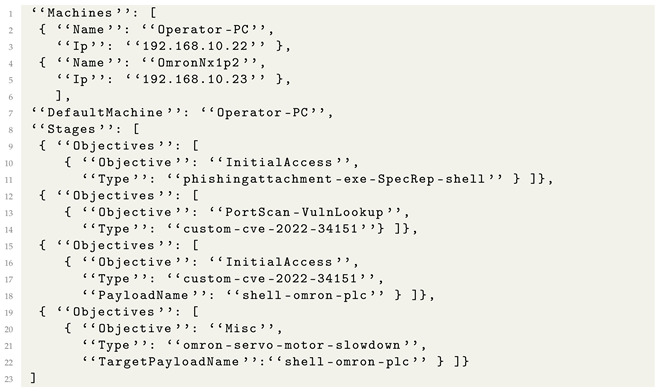



## 5. Discussion

SpecRep defines a well-known and extendable set of attacker objectives that need to be achieved. For each objective, we create a series of possible implementations of actions that can be used to achieve the objective. We base our implementations on forensics reports describing advanced persistent threats or advanced malware. For each forensics report, we create metalanguage files to describe the attack’s objectives and timeline.

The creation of new implementations for actions is an ongoing process. Each time we add a new way to achieve an objective, SpecRep can automatically include it in all previously described attack scenarios by recompiling the specifications. This process models the real-life advances of attacker TTPs (Tactics, Techniques, and Procedures) and ensures that an organization is prepared to deal with both known versions of an attack and plausible future versions of the same attack. This dynamic updating mechanism is crucial in maintaining the relevance and effectiveness of an adversary emulation like SpecRep in the face of rapidly evolving cyber threats.

To ensure the robustness and accuracy of our system, we tested our system on virtual machines that simulate workstations, IoT devices, and Industrial Control Systems. Each machine needs to be previously created and configured. We develop custom emulation software to simulate interactions with physical devices. This allows us to create a controlled environment where we can observe the behavior of the simulated attacks and refine our implementations based on real-world data.

The virtual environments created for testing mimic the complexity and diversity of real-world networks. By including IoT devices and Industrial Control Systems, we address the increasing convergence of IT and OT (Operational Technology) environments, which is a growing concern for cybersecurity professionals.

The examples we previously presented are some parts of real-world attacks that we shortened and altered so as to be easy for the reader to follow. For Mirai, we used the description found in [27] and simplified the attack flow to use three exploits that can be logically chained. For APT 28, we used Responder due to what we read in the US indictment [42]. We presented a scenario with EternalBlue as an entry point based on other information from the MITRE page [43]. Finally, for Chernovite Badomen, we simplified information from [40].

We compare our results with three of the most popular attack simulation tools: SafeBreach, Picus Security Control Validation, and MITRE Caldera.

SafeBreach is a cybersecurity platform that focuses on continuous security validation through breach and attack simulation based on MITRE ATT&CK. The framework is closed-source but offers users customization options via SafeBreach Studio [44,45]. Users can manually create new attack plans based on existing implementations found in the SafeBreach Hacker’s Playbook, a continuously updated library of attack simulations used within the SafeBreach platform [46].

Picus Security Control Validation [47] is another commercial BAS tool that, like SafeBreach, aligns with MITRE ATT&CK. Picus uses agents installed on hosts to simulate attack actions from the Threat Library. Operators can define various templates of attacks via the tool’s graphical user interface or REST API [48]. These templates can be filled in with various predefined threats created by Picus engineers.

MITRE Caldera is an open-source automated adversary emulation system. The core components used by the framework are “attacks”, “planners”, and “agents”.

“Attacks” refer to specific actions or techniques that an adversary might use to achieve their objectives within a target environment. To add new attacks to MITRE Caldera, you generally need to first understand the attack technique. If the new attack requires capabilities that are not available in existing Caldera plugins, one can create a new plugin by writing Python code to define the behavior of this plugin and, finally, one needs to define the attack in YAML [49].

“Planners” are responsible for orchestrating the sequence of attacks during an operation. They determine the order in which attacks should be executed based on a given strategy or objective. Planners take into account the current state of the target environment, the success or failure of previous attacks, and the overall goals of the operation. Adding a new planner requires writing a Python implementation and a YAML definition of the planner [50].

“Agents” [51] are software components deployed on target systems within the network to execute the attacks planned by the operation. These agents communicate with the CALDERA server, receiving instructions on which attacks to execute and reporting back on the success or failure of those actions. Writing a new agent can be carried out in any programming language as long as the agent respects the C2 communication protocols imposed by Caldera. Because the entire framework is open-source, the default agent named “Sandcat” may be extended as well.

Similarly to SafeBreach, Picus, and Caldera, SpecRep uses agents deployed on systems within the network to perform the attack. Extending SpecRep is similar to Caldera: users can implement new attacker objectives or ways to achieve objectives by writing code and describing the additional capability using a metalanguage file.

The primary advantage of SpecRep over the previously mentioned solutions is its ability to automatically extract information from white papers and generate a series of attack plans. We believe that the ideas behind SpecRep’s attack compiler could be adapted to work with SafeBreach, Picus, or Caldera.

A secondary advantage of SpecRep is its capability to function with incomplete information. Specifying a start objective for initial access and an end objective, such as data exfiltration, will cause the SpecRep attack compiler to generate all paths from initial access to exfiltration. This approach is particularly useful when testing the effectiveness of EDR and XDR solutions, as running all scenarios can reveal potential gaps in visibility.

## 6. Future Work

A possible extension of SpecRep would be to apply the attack compiler for computer forensics, as adversary emulation has been proven to improve such scenarios [52,53]. This can be performed as follows.

Assume that initial forensic evidence found a set of artifacts corresponding to objectives achieved by the attacker. We can translate these artifacts into objectives, compile all possible attack maps that contain these objectives, and present the most likely paths to a forensic investigator. This forensic process may be aided by tools that can perform live queries in the infrastructure and may be further augmented by identifying threat actors and forming playbooks with only those Expected Event Specifications that fit the TTPs used by the identified threat actor.

## 7. Conclusions

In this paper, we explored the development and implications of an adversary emulation framework capable of replicating the Tactics, Techniques, and Procedures (TTPs) of advanced actors within heterogeneous networks protected by security solutions.

Our proposed SpecRep system emulates attacks based on objective specifications rather than a predefined sequence of actions. Within SpecRep, a single metalanguage file is translated automatically into all known combinations that fulfill the specified objectives, significantly expanding testing coverage. Additionally, any new method added to perform an action propagates to all existing attack specifications.

We utilized text processing tools, including large language models, to automatically extract attack specifications from freely available white papers. This allows us to generate new attack specifications soon after an attack becomes public knowledge.

The advantages of our system were demonstrated through the emulation of various attacks, including a Mirai attack on a smart home, an enterprise network attack inspired by APT28, and an attack on industrial infrastructure. These demonstrations underscore the effectiveness and versatility of SpecRep in enhancing the testing and evaluation of security solutions against advanced persistent threats.

Moreover, the relevance of our work extends to the domain of smart cities, where the rapid integration of IoT devices and interconnected systems heightens the risk of sophisticated cyber attacks. By emulating adversary tactics in such environments, SpecRep aids in identifying vulnerabilities and improving the resilience of critical infrastructure, ensuring the safety and security of urban populations in the face of evolving cyber threats.

## Figures and Tables

**Table 1:** Number of attack specifications extracted from white papers for each threat actor.

**APT28**	**APT-29**	**APT-34**	**APT-3**	**APT-40**	**IoT Botnets**
6	4	3	4	3	6
**Carbanak**	**FIN 7**	**Sandworm**	**Turla**	**Wizard Spider**	**ICS**
3	4	3	6	4	8

## Data Availability

The authors do not have permission to disclose the data.
